# Efficacy of the 28S rDNA barcode in differentiating Caribbean octocorals

**DOI:** 10.3897/BDJ.12.e140454

**Published:** 2024-12-27

**Authors:** Sloan R Senofsky, Isabel Zamudio, Brittany Pan, Catherine S McFadden

**Affiliations:** 1 Harvey Mudd College, Claremont, United States of America Harvey Mudd College Claremont United States of America

**Keywords:** gorgonian, eDNA, species delimitation, mtMutS

## Abstract

The ecological landscape of Caribbean reefs is rapidly changing as octocorals fill the void left by declining scleractinian populations. Effective molecular barcodes are necessary to accurately identify these octocorals and monitor this shifting ecosystem. We tested the efficacy of the *28S rDNA* as a barcode compared to the most commonly used *mtMutS* barcode on a collection of octocorals from across the Caribbean. Based on pairwise genetic distance values, *28S* appeared to be more effective at differentiating species within the families Plexauridae and Gorgoniidae, while *mtMutS* was slightly more effective at distinguishing species of Pterogorgiidae. However, the standard *28S* rDNA primers did not amplify all species as effectively as *mtMutS*, especially those belonging to the genus *Eunicea*. A shorter *28S* barcode developed for eDNA applications distinguished species as effectively as the complete *28S* barcode.

## Introduction

Worldwide degradation of coral reefs has been well documented as a result of global warming trends along with local contributions of eutrophication, siltation and harmful fishing practices (Hoegh-Guldberg et al. 2007, Hoegh-Guldberg et al. 2017). Caribbean reefs have experienced an increase in water temperatures that has been correlated with disproportionately negative effects on scleractinian populations and a concomitant increase in gorgonian populations with possible extensive ecological consequences (Tsounis and Edmunds (2017), Lasker et al. (2020)). In light of the sheer abundance, diversity and contribution to reef structure that octocorals provide in the Caribbean, tracking changes in this transitioning ecosystem's biodiversity is a critical component for better informing environmental conservation efforts and policy prescriptions aimed at protecting Caribbean reefs (Lasker and Coffroth 1983, Etnoyer et al. 2010, Sánchez 2016, Tsounis et al. 2018).

Many gorgonians within the Caribbean are endemic and closely related, resulting in an abundance of sister species that cannot reliably be distinguished visually in photographs due to their similarities in gross morphology (Wirshing et al. 2005, Tsounis and Edmunds 2017, Lasker et al. 2020). Visual differentiation is further complicated by observed phenotypic plasticity of octocorals within the Caribbean, both in colony growth form (Prada et al. 2008, Bilewitch et al. 2010) and in the morphology of the microscopic skeletal elements (sclerites) on which most species-level taxonomy is based (West et al. 1993, West 1997). The ability to resolve populations at the species level is essential when dissimilarities in community structures are primarily at the species level and not detected with genus-level taxonomic resolution (Tsounis et al. 2018). Molecular barcodes have been explored as a tool to effectively distinguish species within the Caribbean in order to detect these meaningful differences in populations, but, to this point, no single-locus barcode has been shown to differentiate species of octocorals with a high success rate (Baco and Cairns 2012, Morín et al. 2019). The current array of barcodes that have been explored include the mitochondrial genes *COI*, *mtMutS*, NADH-dehydrogenase subunits 2 and 6 and *16S rDNA*, along with nuclear markers, such as *ITS* and *28S rDNA* (Baco and Cairns 2012, McFadden et al. 2014, Morín et al. 2019). Mitochondrial markers are subject to slow rates of evolution in octocorals which presents a challenge in searching for a barcode that can effectively differentiate closely-related species (Shearer et al. 2002, France and Hoover 2002, Hellberg 2006, Shearer and Coffroth 2008, McFadden et al. 2011, Bilewitch and Degnan 2011). Morín et al. (2019) compared the utility of *COI*, *16s rDNA*, *nad2*, *nad6* and *mtMutS* molecular barcodes for species discrimination in Caribbean octocorals. The markers they tested appear to be ineffective at differentiating the majority of species using genetic distance thresholds, especially those species in the family Plexauridae. As this is one of the most speciose families of Caribbean octocorals within which species are most difficult to distinguish morphologically, identification of a species-specific barcode merits further investigation.

The nuclear ribosomal molecular barcode, *28S rDNA*, has been shown to exhibit interspecific variation equal to or exceeding that of mitochondrial markers within octocoral samples collected in the Indo-Pacific (McFadden et al. 2014) and more effectively discriminates species in some taxa of octocorals (Benayahu et al. 2012, Xu et al. 2023, Baena et al. 2024), particularly when combined with the mitochondrial barcode *mtMutS* (Quattrini et al. 2019). The *28S rDNA* barcode has been utili**z**ed for phylogenetic reconstruction of Eastern Pacific gorgonians in the family Gorgoniidae, which resulted in disagreement in tree topology between *mtMutS* and *28S* (Ament-Velásquez et al. 2016, Soler-Hurtado et al. 2017). This *28S* barcode has yet to be tested on shallow-water gorgonians in the Caribbean.

Here, our objectives were to determine if the *28S* marker was better able to distinguish species within families of Caribbean gorgonians and to bioinformatically assess the relative effectiveness of a shorter fragment of *28S rDNA* that has been proposed as a marker for anthozoan eDNA (McCartin et al. 2024).

## Material and methods

Octocorals were sampled using SCUBA (depth < 15 m) at Bocas del Toro, Panama in 2006 and in the Florida Keys, USA in 2019. Pieces of branches (< 10 cm) were removed from colonies using scissors or wire cutters. Small pieces (< 1 cm) of tissue were preserved in 95% ethanol for DNA and the remainder of the sample was dried. Vouchers have been deposited at the US National Museum of Natural History, Smithsonian Institution (NMNH) and Naturalis Biodiversity Center, Leiden, Netherlands (formerly Rijksmuseum van Natuurlijke Historie, RMNH) (Suppl. material 1). Samples were identified to species by comparing gross colony morphological features (e.g. branching pattern, calyx structure, polyp aperture) and sclerite forms to published accounts, in particular Bayer (1961). Tissue from different regions of the colony (e.g. surface, interior, polyps) was digested in household bleach to extract sclerites that were viewed using light microscopy at 10-40x. Morphological IDs were corroborated in consultation with several taxonomic experts, including L.P. van Ofwegen, J.A. Sánchez and H.R. Lasker; samples from the Florida Keys were identified to species during the Caribbean Octocoral Workshop held at Keys Marine Laboratory in June 2019.

The DNeasy Blood & Tissue Kit (Qiagen, Inc.) was used to isolate DNA from ethanol-preserved tissue samples. Fragments of nuclear *28S rDNA* and mitochondrial *mtMutS* were amplified via polymerase chain reaction (PCR) and sequenced using published primers and protocols (McFadden et al. 2011, McFadden and van Ofwegen 2013). Following amplification and sequencing, all specimens were categorised as: (a) successfully amplified with a readable sequence or (b) successfully amplified with an unreadable sequence or failed to amplify. Sequences with readable chromatograms were assembled into contigs and edited using DNAStar Lasergene.

*28S rDNA* and *mtMutS* sequences were aligned using ClustalW and MUSCLE methods, respectively, within MEGA7 (Kumar et al. 2016). Alignments were verified and adjusted by hand if necessary. Pairwise genetic distances (uncorrected p) between specimens were calculated using MEGA7; mean pairwise genetic distances were calculated amongst genera and amongst species within families. In addition, the ends of aligned *28S* sequences were trimmed to leave only the segment that is amplified by recently published eDNA primers (McCartin et al. 2024) and pairwise genetic distances were calculated separately for that fragment.

## Data resources

All DNA sequences have been deposited to GenBank; accession numbers can be found in Suppl. material 1.

## Results

*28S rDNA* sequences (~ 765 bp) were obtained for 93 individuals across 12 genera and 30 species plus an additional 31 individuals identified only to the genus level. (Table 1; Suppl. material 1). The *28S* primers (28S-Far, 28S-Rar; McFadden and van Ofwegen (2013)) successfully sequenced 78.2% of samples that were identified to species. In approximately 35% of the unsuccessful sequencing attempts, amplicons were obtained, but the sequences were unreadable. *mtMutS* sequences (~ 735 bp) were successfully obtained for 105 individuals across 12 genera and 29 identifiable species (Table 1). *mtMutS* was successfully amplified in 84.4% of samples that were identified to species; seven specimens (two *Carijoariisei*, three *Muriceopsisflavida* and two unidentified *Antillogorgia*) were only amplified using the mut2761F and mut3270R primers (Reijnen et al. 2014) that yielded a truncated barcode (~ 450 bp).

**Figure 1.**
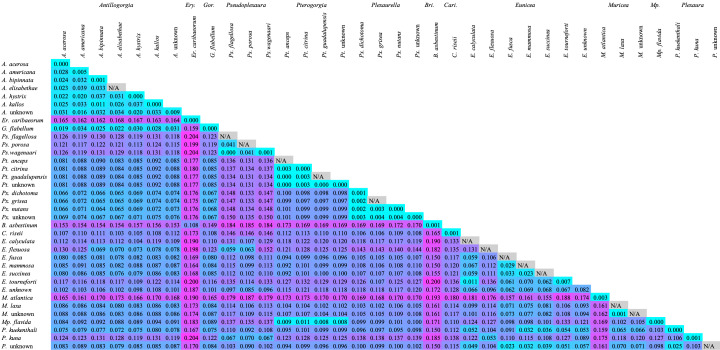
Heatmap of the mean genetic distances (uncorrected p) between species of Caribbean octocorals using the *28S rDNA* barcode. Cell colours: cyan most similar, magenta most different. N/A: Sample size insufficient to calculate intra-species genetic distances.

**Figure 2.**
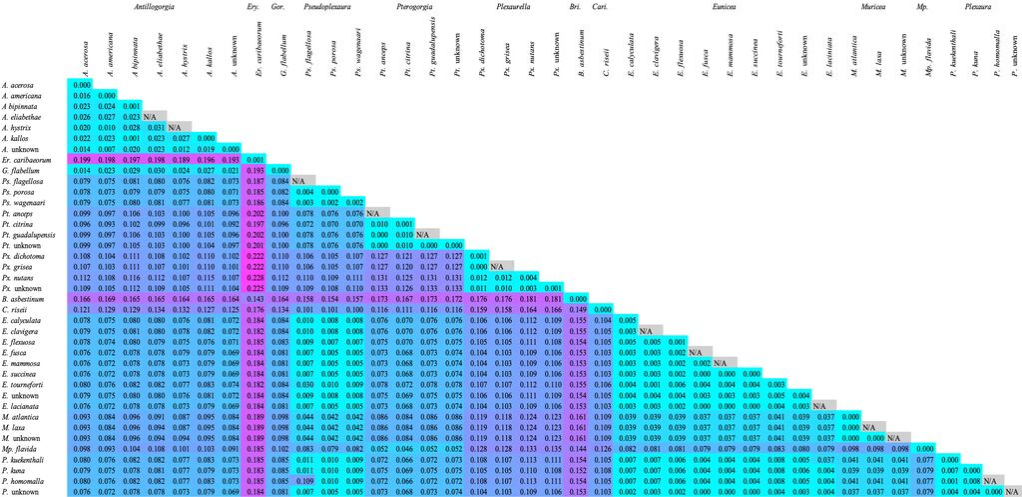
Heatmap of the mean genetic distances (uncorrected p) between species of Caribbean octocorals using the *mtMutS* barcode. Cell colours: cyan most similar, magenta most different. N/A: Sample size insufficient to calculate intra-species genetic distances.

*28S rDNA* displayed greater genetic variability between genera, which was evident in the mean pairwise genetic distances. The average intergeneric genetic distance amongst *28S* sequences was 0.1122 (SD = 0.0364, n = 567) (Fig. 1), while amongst *mtMutS*, it was only 0.0881 (SD = 0.0464, n = 660) (Fig. 2).

Intraspecific genetic distances within all families were greater on average when utilising *28S*, with distances that ranged from 0.0000 to 0.0071 with a mean of 0.0011 (SD = 0.0042, n = 18) (Fig. 1), compared to those at *mtMutS*, which ranged from 0.0000 to 0.0054 with a mean of 0.0008 (SD = 0.0013, n = 20) (Fig. 2). Similarly, interspecific genetic distances amongst species in the same family were greater on average when utilising *28S* compared to *mtMutS*. The mean interspecific genetic distance in *28S* was 0.1094 (SD = 0.0435, Min = 0.0000, Max = 0.2040, n = 435) (Fig. 1), while the mean interspecific genetic distance in *mtMutS* was only 0.0760 (SD = 0.1991, Min = 0.0000, Max = 0.1991, n = 529) (Fig. 2).

**Figure 3.**
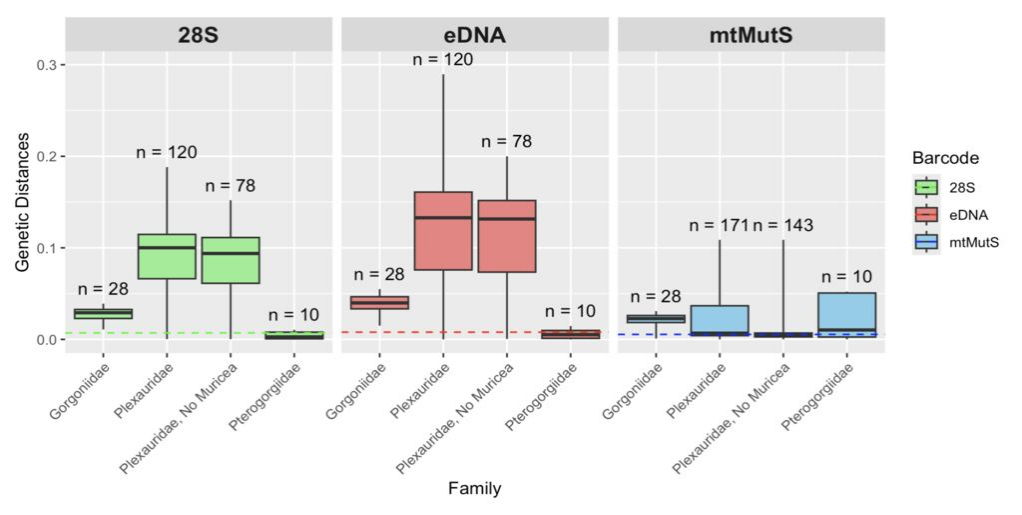
Mean pairwise genetic distances between species of Caribbean octocorals within the families Gorgoniidae, Plexauridae, Pterogorgiidae and Plexauridae without *Muricea*. Broken lines indicate the maximum mean intraspecific distance recorded for each barcode. n = number of mean pairwise interspecific genetic distances.

Defining the threshold for successful differentiation as the mean pairwise interspecific genetic distance being greater than the maximum intraspecific genetic distance for the barcode, *28S* more effectively differentiated species in the family Gorgoniidae, differentiating 100% of pairwise comparisons (n = 28), while *mtMutS* had a 96% success rate (n = 28) (Fig. 3). This trend was also observed within the family Plexauridae where *28S* successfully differentiated 98% of pairwise comparisons including *Muricea* (n = 120) and 99% excluding *Muricea* (n = 78), while *mtMutS* only differentiated 58% (n = 171) including *Muricea* and 61% without *Muricea* (n = 143) (Fig. 3). However, *28S* was marginally less successful than *mtMutS* at differentiating species in the family Pterogorgiidae with the successful differentiation of 6 out of 10 comparisons, while *mtMutS* succeeded in 7 out of 10 comparisons (Fig. 3).

**Figure 4.**
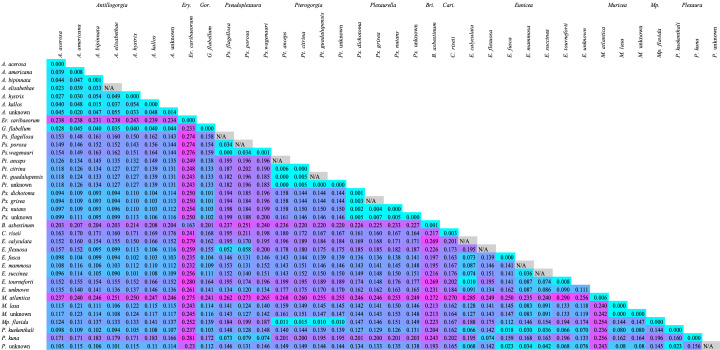
Heatmap of the mean genetic distances (uncorrected p) between species of Caribbean octocorals using the *28S rDNA* sequence fragment developed for eDNA applications (McCartin et al. 2024). Cell colours: cyan most similar, magenta most different. N/A: Sample size insufficient to calculate intra-species genetic distances.

The eDNA barcode had a greater intraspecific genetic distance range from 0.0000 to 0.0079 with a mean value of 0.0016 (SD = 0.0027, n = 18) (Fig. 4). The eDNA barcode had uniformly greater mean interspecific genetic distances than *28S* with an average of 0.1536 (SD = 0.0625, Min = 0.0000, Max = 0.2896, n = 435). Additionally, the eDNA barcode performed identically to the complete *28S* barcode in regard to the percentage of interspecific pairwise comparisons that were greater than the maximum intraspecific genetic distance (Fig. 3).

## Discussion

The *28S rDNA* barcode appears to be more successful at differentiating genera and species within the families Plexauridae and Gorgoniidae, while *mtMutS* was marginally more effective at differentiating species belonging to the family Pterogorgiidae (Fig. 3). However, only 10 interspecific comparisons were utilised in the Pterogorgiidae analysis, which lends uncertainty to the significance and accuracy of this observed discrepancy in performance. Further investigation involving a greater sample size is necessary for the accurate comparison of barcode efficacy within this family. While some previous studies of other octocoral families have concluded that *28S* better discriminates species than *mtMutS* (e.g. Benayahu et al. (2012), Xu et al. (2023), Baena et al. (2024)), other studies have found no difference or a lack of congruence between the two barcodes (Quattrini et al. 2019, Quattrini et al. 2022). While *28S* seems to be a superior barcode for discrimination of Caribbean plexaurids and gorgoniids, that result cannot be assumed to hold for all octocoral taxa.

Morín et al. (2019) evaluated the efficacy of five mitochondrial barcodes in Caribbean gorgonians. Using both genetic distances and haplotype networks, they concluded that all of the barcodes they tested could effectively differentiate the majority of gorgonian genera, with the exception of the plexaurids *Eunicea*, *Plexaura* and *Pseudoplexaura*. The *mtMutS* barcode was the most effective at distinguishing species in those genera, but some species of *Eunicea*, *Plexaura* and *Pseudoplexaura* shared the same haplotypes. Our results align with Morín's and support their conclusion that *mtMutS* is an unreliable tool for species differentiation within Plexauridae. A noteworthy difference amongst the studies is that Morín et al. (2019) used the absolute number of base pair differences as their genetic distance metric, while this study uses uncorrected p (i.e. % of base pair differences). To accurately compare results, their bp differences can be converted to genetic distances by dividing base pair differences by the total barcode length.

Based on amplification success, which is the percentage of samples that successfully amplified and yielded readable chromatogram sequences, *28S* and *mtMutS* primers were comparable for the majority of specimens that were tested. An observed shortcoming of the *28S* barcode, however, is its limited ability to amplify specimens within the genus *Eunicea*. Furthermore, these *28S* sequences required significantly more care when proofreading to resolve ambiguities in the chromatograms. The unclear chromatogram results could be due to secondary structures in the *28S* sequence interfering with the amplification or sequencing process or to the presence of intra-individual polymorphisms in *28S* within a species. Further study will be necessary to confirm either of these hypotheses.

Since the effectiveness of the eDNA barcode was analysed bioinformatically by truncating the longer *28S* sequence amplified using the standard primers, questions remain regarding the efficacy of the eDNA primers for amplification, in particular in *Eunicea*. Further testing of the eDNA primers in vitro will be necessary to determine if they increase the amplification success rate and minimise sequencing artefacts in that genus and other species that amplified inconsistently.

While this study utilised a genetic-distance approach that established the threshold for differentiating species as the maximum intraspecific genetic distance, further investigation of these barcodes could identify pure characteristic attributes that can be utilised to differentiate samples (i.e. a nucleotide that is present in all individuals of a species and absent in other species). The presence of pure diagnostic attributes, an attribute or combination of attributes that differentiate samples with a high degree of certainty, could allow for the character-based differentiation of samples that are inadequately differentiated using an intraspecific genetic distance threshold (DeSalle et al. 2005, McFadden et al. 2011). Larger sample sizes with a greater number of individuals belonging to the same species will be required to establish reliable character-based barcodes for the majority of these species. Furthermore, with a larger number of specimens belonging to the same species, we will be better able to quantify intraspecific variation in these barcode markers.

A larger-scale analysis of Caribbean gorgonians would benefit from the integration of a broader range of molecular barcodes in addition to morphological identification at the colony level and sclerite level to increase confidence in identifications.

## Supplementary Material

71896D17-66F4-520F-8225-131BFB9AEBFE 10.3897/BDJ.12.e140454.suppl1 Supplementary material 1 Panama and Florida Keys Octocoral Identification Information Data type Museum voucher and GenBank accession numbers File: oo_1199183.xlsx https://binary.pensoft.net/file/1199183 Sloan R Senofsky, Isabel Zamudio, Brittany Pan, Catherine S McFadden

## Figures and Tables

**Table 1.** Number of individuals collected for each species of Caribbean gorgonian and success rate for amplification with each barcode marker. %: percentage of individuals that were successfully amplified and yielded readable sequences; num: total number of samples that we attempted to amplify via PCR. Species with < 100% amplification success are in bold for clarity. Results shown only for samples that were identified to species. *Some specimens amplified using mut2761F-mut3270R primers only.

	*mtMutS*		*28S rDNA*	
	%	num	%	num
*Antillogorgiaacerosa* (Pallas, 1766)	100	8	100	8
*Antillogorgiaamericana* (Gmelin, 1791)	**83**	12	100	12
*Antillogorgiabipinnata* (Verrill, 1864)	**79**	14	**93**	14
*Antillogorgiaelisabethae* (Bayer, 1961)	100	1	100	1
*Antillogorgiahystrix* (Bayer, 1961)	100	1	100	2
*Antillogorgiakallos* (Bielschowksy, 1918)	100	2	100	2
*Briareumasbestinum* (Pallas, 1766)	100	7	100	7
*Carijoariisei* (Duchassaing & Michelotti, 1860)	100	3*	**67**	6
*Erythropodiumcaribaeorum* (Duchassaing & Michelotti, 1860)	100	2	**50**	4
*Euniceaasperula* Milne Edwards & Haime, 1857	**0**	2	**0**	2
*Euniceacalyculata* (Ellis & Solander, 1786)	**67**	3	**25**	4
*Euniceaclavigera* Bayer, 1961	100	1	N/A	N/A
*Euniceaflexuosa* (Lamouroux, 1821)	**75**	4	**50**	2
*Euniceafusca* Duchassaing & Michelotti, 1860	100	1	100	1
*Eunicealaciniata* Duchassaing & Michelotti, 1860	100	1	**0**	1
*Euniceamammosa* Lamouroux, 1816	**50**	2	100	1
*Euniceasuccinea* (Pallas, 1766)	**86**	7	**20**	5
*Euniceatayrona* Sánchez, 2009	**0**	2	**0**	1
*Euniceatourneforti* Milne Edwards & Haime, 1857	**80**	5	**50**	4
*Gorgoniaflabellum* Linnaeus, 1758	100	2	100	2
*Muriceaatlantica* (Riess in Kükenthal, 1919)	100	2	100	2
*Muricealaxa* Verrill, 1864	100	1	100	1
*Muriceopsisflavida* (Lamarck, 1815)	**83**	6*	100	6
*Plexaurahomomalla* (Esper, 1794)	100	1	**0**	1
*Plexaurakuekenthali* Moser, 1921	100	2	100	2
*Plexaurakuna* Lasker, Kim & Coffroth, 1996	100	4	100	3
*Plexaurelladichotoma* (Esper, 1788)	**83**	6	100	5
*Plexaurellagrisea* Kunze, 1916	**33**	3	**50**	2
*Plexaurellanutans* (Duchassaing & Michelotti, 1860)	100	2	100	2
*Pseudoplexauraflagellosa* (Houttuyn, 1772)	100	1	100	1
*Pseudoplexauraporosa* (Houttuyn, 1772)	100	4	**20**	5
*Pseudoplexaurawagenaari* (Stiasny, 1941)	**83**	6	100	5
*Pterogorgiaanceps* (Pallas, 1766)	100	1	100	1
*Pterogorgiacitrina* (Esper, 1792)	100	2	100	2
*Pterogorgiaguadalupensis* Duchassaing & Michelin, 1846	100	1	100	1
